# A phase 3 randomized, double-blind, placebo-controlled study to evaluate the efficacy and safety of sarilumab in patients with giant cell arteritis

**DOI:** 10.1186/s13075-023-03177-6

**Published:** 2023-10-16

**Authors:** Wolfgang A. Schmidt, Bhaskar Dasgupta, Jennifer Sloane, Angeliki Giannelou, Yuqing Xu, Sebastian H. Unizony, Sarah L. Mackie, Miguel A. Gonzalez-Gay, Robert Spiera, Kenneth J. Warrington, Peter M. Villiger, Michael C. Nivens, Bolanle Akinlade, Yong Lin, Frank Buttgereit, John H. Stone

**Affiliations:** 1 Medical Centre for Rheumatology Berlin-Buch, Immanuel Krankenhaus Berlin, Lindenberger Weg 19, Berlin, 13125 Germany; 2 Southend University Hospital, Mid and South Essex NHS Foundation Trust, Essex, UK; 3 Sanofi, Cambridge, MA USA; 4 Regeneron Pharmaceuticals, Inc, Tarrytown, NY USA; 5 Sanofi, Bridgewater, NJ USA; 6 Massachusetts General Hospital, Harvard Medical School, Boston, MA USA; 7 Leeds Institute of Rheumatic and Musculoskeletal Medicine, University of Leeds, Leeds, UK; 8 Leeds Biomedical Research Centre, Leeds Teaching Hospitals NHS Trust, Leeds, UK; 9 Rheumatology Division, IIS-Fundación Jiménez Díaz, Madrid, Spain; 10 University of Cantabria, IDIVAL, Santander, Spain; 11 Department of Medicine, Hospital for Special Surgery, New York, NY USA; 12 Division of Rheumatology, Department of Medicine, Mayo Clinic, Rochester, MN USA; 13 Rheumatology and Clinical Immunology, Medical Center Monbijou, Bern, Switzerland; 14 Department of Rheumatology and Clinical Immunology, Charité Universitätsmedizin Berlin, Berlin, Germany

**Keywords:** Sarilumab, Giant cell arteritis, Glucocorticoids, Interleukin-6, Sustained remission

## Abstract

**Background:**

Giant cell arteritis (GCA) is primarily treated with glucocorticoids (GCs), which have substantial toxicity. Tocilizumab, an interleukin-6-receptor inhibitor (IL-6Ri), showed beneficial effects in GCA, leading to its approval. This study investigated the efficacy and safety of sarilumab (another IL-6Ri) in GCA*.*

**Methods:**

This Phase 3, double-blind study comprised a 52-week treatment period and a 24-week follow-up phase. Eligible GCA patients were randomized to receive sarilumab 200 mg (SAR200 + 26W) or 150 mg (SAR150 + 26W) with a 26-week GC taper, or placebo with a 52-week (PBO + 52W) or 26-week (PBO + 26W) GC taper. The primary efficacy endpoint was sustained remission (SR) at week 52. Additional endpoints were SR at week 24, cumulative GC dose, and safety. The study was discontinued prematurely due to protracted recruitment timelines, because of the impact of COVID-19. Therefore, only descriptive statistics were summarized.

**Results:**

Of the planned 360 subjects, only 83 were randomized and 36 were included in the week 52 analysis. At week 52, 46% (*n* = 6/13) of patients in SAR200 + 26W, 43% (*n* = 3/7) in SAR150 + 26W, 30% (*n* = 3/10) in PBO + 52W, and 0 (*n* = 0/6) in PBO + 26W taper groups achieved SR. Sensitivity analyses, excluding acute-phase reactants from the SR definition, showed similar results for SAR groups, but 60% (*n* = 6/10) in PBO + 52W and 17% (*n* = 1/6) in PBO + 26W taper groups achieved SR at week 52. Similar findings were noted at week 24. The proportions of patients who adhered to GC taper from week 12 through week 52 in each group were as follows: 46% (*n* = 6/13, SAR200 + 26W), 43% (*n* = 3/7, SAR150 + 26W), 60% (*n* = 6/10, PBO + 52W), and 33% (*n* = 2/6, PBO + 26W). The median actual cumulative GC dose received in the SAR200 + 26W group was lower than other groups. Most patients (80–100%) experienced treatment-emergent adverse events, with similar incidences reported across groups.

**Conclusions:**

Owing to the small sample size due to the early termination, it is difficult to draw clear conclusions from this study. There were no unexpected safety findings.

**Trial registration:**

ClinicalTrials.gov NCT03600805. Registered on July 26, 2018.

**Supplementary Information:**

The online version contains supplementary material available at 10.1186/s13075-023-03177-6.

## Background

Giant cell arteritis (GCA) is the most common primary systemic vasculitis in people above 50 years of age and is more frequently reported in Caucasians and in females [1, 2]. More than 3 million people are expected to be diagnosed with GCA by 2050 in Europe, North America, Australia, and New Zealand [3].

Patients with GCA may present with constitutional, polymyalgia, or cranial symptoms. Ischemic complications, such as anterior ischemic optic neuropathy or stroke, may occur if treatment is delayed [4, 5]. Early identification and successful treatment are, therefore, critical in preventing permanent vision loss and other significant morbidities.

Glucocorticoids (GCs) are an integral part of the treatment of GCA [6–9]. However, long-term GC use is associated with adverse effects including diabetes mellitus, arterial hypertension, coronary heart disease, weight gain, infections, osteoporosis, cataract, and depression. Relapses are also common in GCA when GC doses are reduced. Further, it is now recognized that treatment with GCs alone leads to both inadequate rates of GC-free remission and an unacceptably high incidence of treatment-related adverse effects with long-term use [6–13]. For these reasons, treatment strategies to maintain GCA in remission and decrease GC exposure are needed.

The role of interleukin-6 (IL-6) is well documented in the pathogenesis of GCA, with increased IL-6 levels found in the temporal artery tissues of patients with active disease. In addition, circulating IL-6 is elevated in untreated GCA and correlates with poor clinical response [14, 15]. Tocilizumab (TCZ), an interleukin-6-receptor inhibitor (IL-6Ri), demonstrated significant efficacy in Phase 2 and 3 trials [16, 17], leading to its approval in the USA and Europe for the treatment of patients with GCA [18–20]. However, there is currently no other drug that has unequivocally demonstrated efficacy for the treatment of GCA, thereby arguing for the need for treatment alternatives.

Sarilumab, another IL-6Ri, is approved worldwide for the treatment of rheumatoid arthritis (RA) and for polymyalgia rheumatica (PMR) in the USA [21]. Sarilumab is known to have high affinity for IL-6 receptor alpha [22] and was investigated as a treatment option for patients with GCA.

This study (NCT03600805) was designed to evaluate the efficacy and safety of sarilumab in patients with active GCA over 52 weeks. Although the protracted recruitment timelines exacerbated by the pandemic of coronavirus disease-2019 (COVID-19) led to premature discontinuation of the trial by the sponsor, we report the available findings herein.

## Methods

### Study design and population

**Fig. 1:**
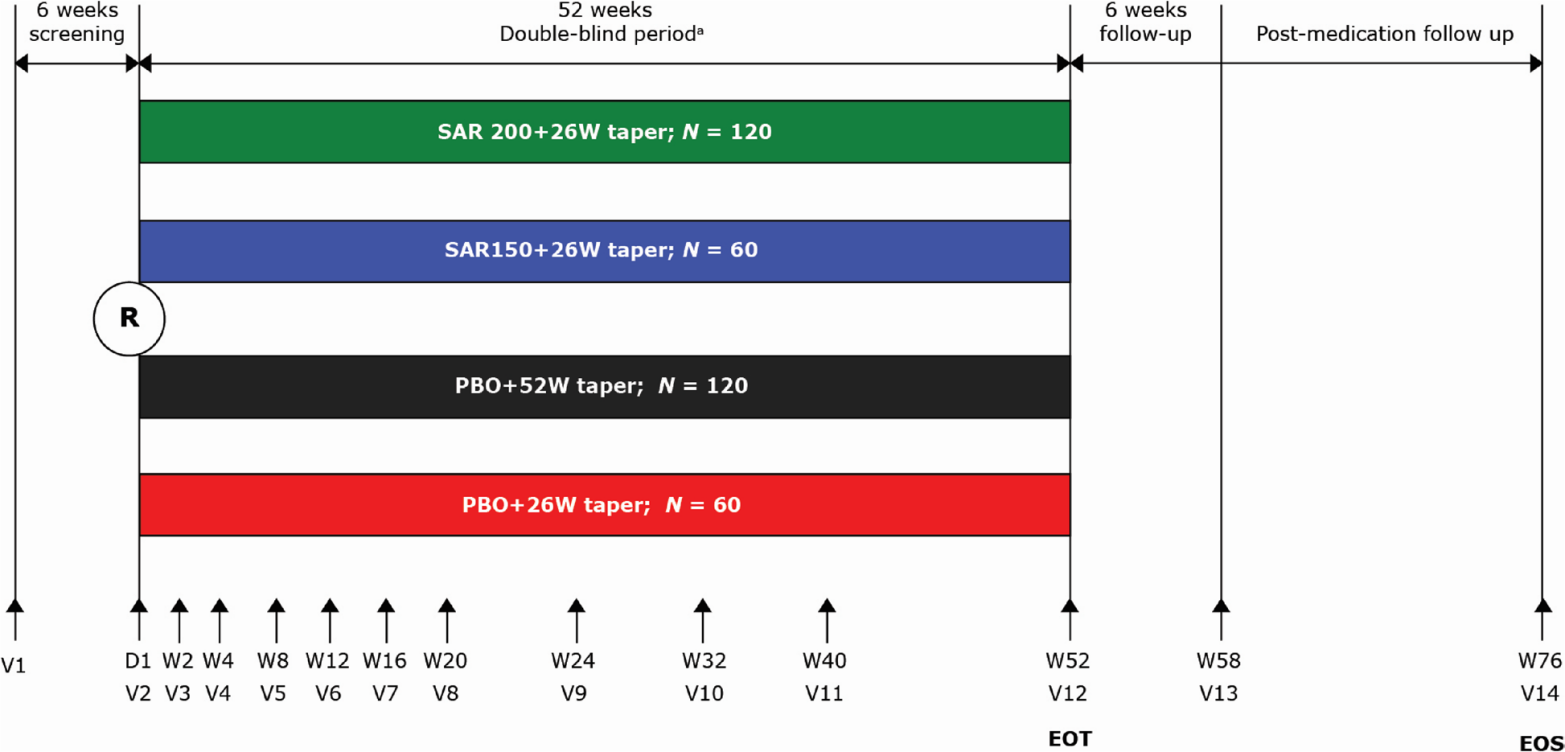
Study design. ^a^Patients experiencing a disease flare may be rescued with open-label treatment as per Investigator’s judgment during the study treatment period. D, day; EOS, end of study; EOT, end of treatment; *N*, number of patients; PBO, placebo; R, randomization; SAR150/200, sarilumab 150/200 mg; V, visit; W, week

This was a Phase 3, multicenter, randomized, double-blind, placebo-controlled, 52-week study with a 24-week post-treatment follow-up phase (NCT03600805) (Fig. 1). The study was conducted according to the principles defined in the Declaration of Helsinki and Good Clinical Practices. All participating investigators obtained full ethics or institutional review board approval according to their local regulations. Written informed consent was obtained from all participating patients.

Patients were included in the study if they had a diagnosis of GCA according to the following criteria: ≥ 50 years of age; history of erythrocyte sedimentation rate (ESR) ≥ 50 mm/h (or C-reactive protein [CRP] > 25 mg/L); unequivocal cranial symptoms of GCA or PMR; and presence of at least one of the following: temporal artery biopsy (TAB) revealing features of GCA, evidence of large-vessel vasculitis by angiography or cross-sectional imaging (angiography, computed tomography angiography [CTA], magnetic resonance angiography [MRA], or positron emission tomography-computed tomography [PET-CT], ultrasound, etc.). Patients either had new-onset active GCA (diagnosis within 6 weeks of baseline) or refractory active GCA (diagnosis > 6 weeks before baseline and previous treatment with ≥ 40 mg/day prednisone [or equivalent] for at least consecutive 2 weeks at a time). The detailed inclusion and exclusion criteria are summarized in Additional file 1 (Table S1).

### Study treatment

Patients with GCA (new onset or refractory) who met the inclusion criteria were randomized in a double-blinded manner to four parallel treatment groups as 2:1:2:1 (Fig. 1) and received sarilumab or matching placebo in combination with a 26-week or 52-week standardized GC (prednisone) taper. This randomization was done via an interactive response technology and stratified by the baseline prednisone dose (< 30 or ≥ 30 mg/day). A dual assessor approach was used to maintain the blind during the treatment period with sarilumab, placebo, and GCs [16]. The treatment groups were as follows:

- *SAR200* + *26W taper*: Sarilumab 200 mg every 2 weeks (Q2W) subcutaneous (SC) with a 26-week taper of GC.
- *SAR150* + *26W taper*: Sarilumab 150 mg Q2W SC with a 26-week GC taper.
- *PBO* + *52W taper*: Sarilumab-matching placebo Q2W SC with a 52-week GC taper.
- *PBO* + *26W taper*: Sarilumab-matching placebo Q2W SC with a 26-week GC taper.

A dose reduction to sarilumab 150 mg was allowed for patients randomized to sarilumab 200 mg in response to elevated liver transaminases (increase in alanine aminotransferase to ≥ 3X upper limit of normal [ULN] to ≤ 5X ULN and bilirubin ≤ 2X ULN), neutropenia (neutrophils ≥ 500/mm^3^ to < 1000/mm^3^), and/or thrombocytopenia (platelets ≥ 50,000 cells/mm^3^ to < 100,000 cells/mm^3^). Once the dose was reduced, no further dose increase was permitted for the remainder of the study treatment period.

To optimize GC treatment prior to randomization, patients received oral prednisone between 20 and 60 mg/day with a starting dose at the investigator’s discretion at the time of randomization (baseline). The taper was initially open label with 1–7 weeks to taper the prednisone dose to 20 mg/day following randomization. Further tapering of the prednisone dose was performed over a period of 45 weeks in a blinded manner (patients received a combination of prednisone and/or prednisone-matching placebo, depending on the assigned treatment group and associated taper regimen) [23]. The total tapering period was 46–52 weeks, depending on the initial prednisone dose at randomization. Please refer to Additional files 2 and 3 (Table S2 and Table S3) for detailed tapering schemes.

If any patient experienced a GCA relapse (flare) per the investigator’s clinical judgment, the per-protocol prednisone taper was stopped, and rescue therapy was initiated. For such patients, GC was started first as rescue therapy, and patients were allowed to continue SC administration of sarilumab or matching placebo. If patients remained symptomatic despite GC rescue therapy, then other treatment options (including non-biological immunosuppressive drugs) could be utilized per protocol.

The total duration of the study was planned for up to 82 weeks, including up to 6 weeks screening, 52-week treatment period (sarilumab or placebo, double-blind phase), and 24 weeks post-treatment follow-up.

### Study assessments

The primary efficacy endpoint was the proportion of patients achieving sustained remission (SR) at week 52, which was defined as meeting all of the following parameters: achievement of disease remission (i.e., resolution of signs and symptoms of GCA and normalization of CRP < 10 mg/L) no later than week 12, absence of disease flare (i.e., either recurrence of signs and symptoms or elevation of ESR attributable to active GCA requiring rescue GC) from week 12 through week 52, normalization of CRP to < 10 mg/L (with an absence of successive elevations to ≥ 10 mg/L) from week 12 through week 52, and successful adherence to prednisone taper from week 12 through week 52. As many patients were not able to complete the study due to the premature termination by the sponsor, an additional endpoint was added: the proportion of patients achieving SR at week 24. Sensitivity analyses, both pre-specified and post hoc, were performed for the primary and additional endpoints, using revised definitions for remission, which excluded acute-phase reactants (APR; pre-specific analysis excluded CRP while post hoc analysis excluded both CRP and ESR).

The secondary endpoints were as follows:

- *Efficacy:* Summary of components of the SR composite measure at week 52 and week 24, total cumulative GC (including prednisone) dose during the treatment period, and time to first GCA flare during the treatment period were measured. In addition, the potential effect of sarilumab on sparing GC toxicity was measured using a glucocorticoid toxicity index (GTI), both as cumulative worsening score (CWS) and aggregate improvement score (AIS) at week 52 and week 24. The composite GTI consists of 9 domains and 31 items that assess the potential side effects of GCs, based on evaluation of body mass index (BMI), glucose tolerance, blood pressure, lipids, bone density, steroid myopathy, skin toxicity, neuropsychiatric toxicity, and infections. The CWS calculates the worsening of GC-associated adverse effects, while the AIS assesses both improvement and worsening [24, 25].
- *Safety:* Adverse events (AEs), laboratory values, and vital signs were assessed throughout the study.
- *Pharmacodynamics (PD):* Change from baseline in CRP was measured through week 52, and changes in IL-6 level and soluble IL-6 receptor (sIL-6R) were measured over time from baseline through week 52.
- *Pharmacokinetics (PK):* Serum functional sarilumab concentrations were determined.

### Sample size determination

Based on the results from the GiACTA trial [16], it was expected that the SR rates at week 52 (i.e., the primary endpoint) would be ~ 54% for the SAR200 + 26W taper group, ~ 18% for the PBO + 52W group, and ~ 14% for the PBO + 26W taper group. A conservative SR rate of ~ 50% was assumed for the SAR150 + 26W taper group. A total sample size of 360 patients (*n* = 120 for SAR200 + 26W, *n* = 60 for SAR150 + 26W, *n* = 120 for PBO + 52W, and *n* = 60 for PBO + 26W taper groups) was chosen to provide at least 90% overall power for between-group comparisons on the primary endpoint. This sample size was also selected to ensure adequate power for sensitivity analyses, excluding APR from the primary endpoint definition. All tests were proposed to be performed at a 0.01 significance level (two-sided).

### Changes in conduct of study

The study enrolled the first patient on 20 Nov 2018 but was prematurely discontinued on 21 Jul 2020 due to protracted recruitment, exacerbated by the COVID-19 pandemic; all randomized patients had to stop their study participation in 12 weeks and have a follow-up visit at 6 weeks following treatment cessation (and no later than 24 Nov 2020). As the majority of enrolled patients had the opportunity to reach the week 24 visit, and it was considered a clinically meaningful timepoint to evaluate response, the following additional endpoints were added at week 24: the proportion of patients achieving SR; components of SR composite measure; and GTI (CWS and AIS).

### Analysis populations

The primary endpoint analysis of the patients who achieved SR at week 52 was limited to the cohort of patients who had an opportunity to complete the 52-week treatment period (i.e., week 52 analysis set). This cohort of patients was defined as the patients with a randomization date prior to 16 Oct 2019.

The intent-to-treat (ITT) population (i.e., all randomized patients) was used for the efficacy analysis at week 24. The safety population included all randomized patients who received at least one dose of the study medication. The PK population consisted of all patients in the safety population with at least one post-dose, non-missing serum sarilumab concentration.

### Statistical analyses

Due to the small sample size, only descriptive summaries by four treatment groups are presented for all baseline characteristics and endpoints. All analyses were performed with SAS Enterprise Guide Version ENGLISH 9.4.

## Results

### Patient disposition

**Fig. 2:**
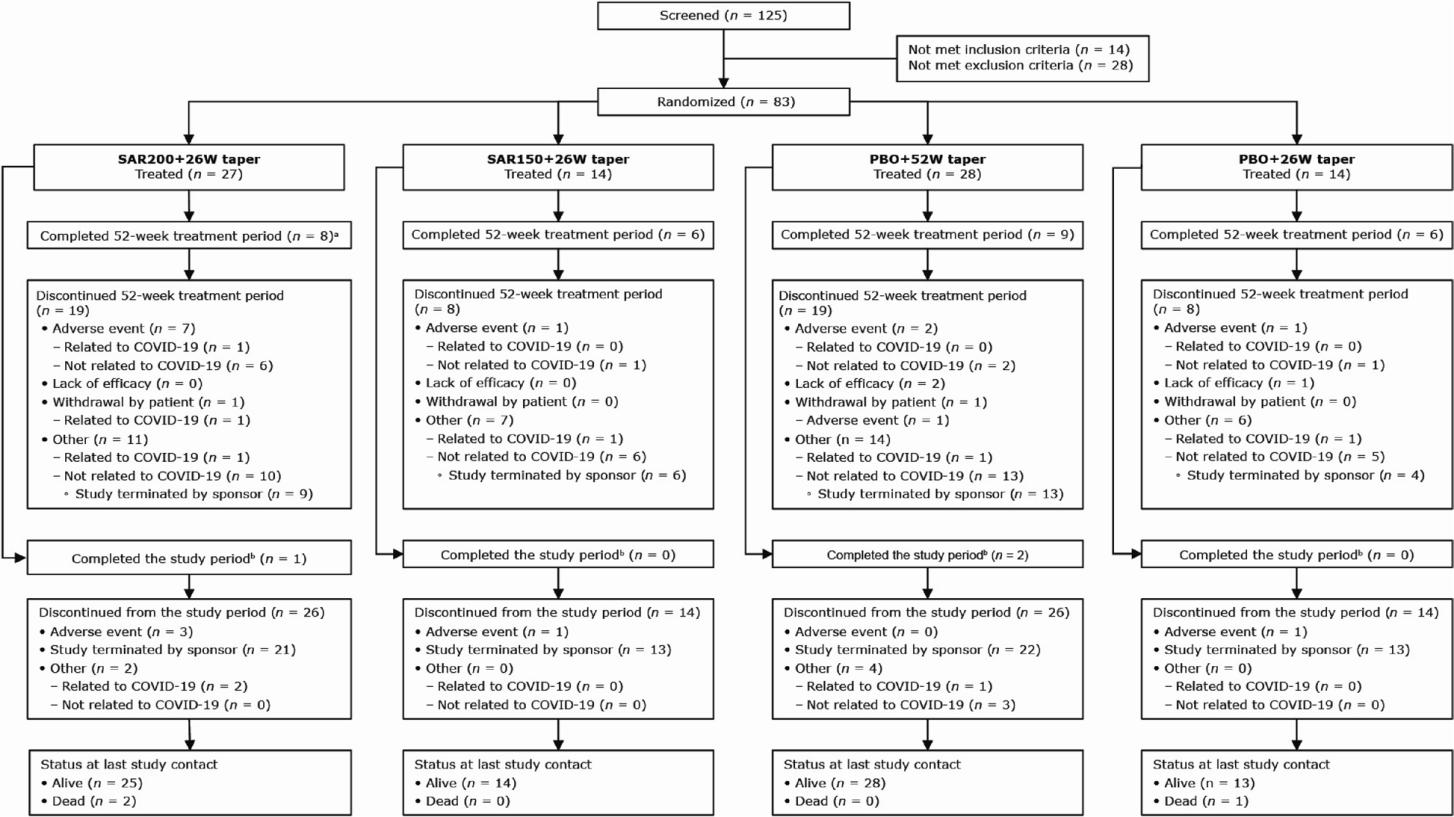
Patient disposition. ^a^One patient completed the study period, including 24 weeks follow-up. ^b^Includes patients who discontinued study treatment but completed scheduled study visits. COVID-19, coronavirus disease-2019; *n*, number of patients; PBO, placebo; SAR150/200, sarilumab 150/200 mg; W, week

Of 125 patients screened, a total of 83 patients were randomized and treated in the study (i.e., ITT population): 27 patients in the SAR200 + 26W taper group, 14 patients in the SAR150 + 26W taper group, 28 patients in the PBO + 52W taper group, and 14 patients in the PBO + 26W taper group (Fig. 2). Approximately two-thirds of these patients did not complete the planned study treatment before 52 weeks (70% [*n* = 19/27] in the SAR200 + 26W taper group, 57% [*n* = 8/14] each in the PBO + 26W and SAR150 + 26W taper groups, and 68% [*n* = 19/28] in the PBO + 52W taper group). The most common reason for treatment discontinuation was the early termination of the study (Fig. 2).

Of the 83 patients, only 36 were randomized prior to 16 Oct 2019 and were included in the week 52 analysis set: 13 patients in the SAR200 + 26W taper group, 7 patients in the SAR150 + 26W taper group, 10 patients in the PBO + 52W taper group, and 6 patients in the PBO + 26W taper group. Of these 36 patients, 29 completed the 52-week treatment period (8 patients in the SAR200 + 26W taper group, 6 patients each in the SAR150 + 26W and PBO + 26W taper groups, and 9 patients in the PBO + 52W taper group).

### Baseline demographics and disease characteristics

The majority of patients were between 65 and 75 years of age, White, and female (Table 1). More than 50% of patients had new-onset GCA across all groups, with similar median prednisone dose at baseline (> 55% of the patients were receiving ≥ 30 mg/day dose). GCA diagnosis was confirmed via imaging in 58 (72%) patients, and via TAB in 23 (28%) patients.

**Table 1:** Patient demographics and disease characteristics at baseline—ITT population

Randomized population (*N* = 83)	SAR200 + 26W taper (*n* = 27)	SAR150 + 26W taper (*n* = 14)	PBO + 52W taper (*n* = 28)	PBO + 26W taper (*n* = 14)
Age (years), mean (SD)	73.4 (8.6)	67.1 (7.9)	71.4 (7.7)	69.5 (5.4)
Female, *n* (%)	23 (85)	13 (93)	22 (79)	9 (64)
Race, *n* (%)				
White	25 (93)	11 (79)	23 (82)	13 (93)
Black or African American	0	1 (7.1)	0	0
Asian	0	0	0	0
Other^a^	2 (7)	2 (14)	5 (18)	1 (7)
BMI (kg/m^2^)				
*N*	27	14	28	13
Mean (SD)	27.2 (5.7)	27.5 (5.2)	25.6 (5.0)	27.8 (4.7)^b^
Smoking, *n* (%)				
Never	16 (59)	12 (86)	20 (71)	11 (79)
Current	2 (7)	1 (7)	4 (14)	2 (14)
Former	9 (33)	1 (7)	4 (14)	1 (7)
GCA, *n* (%)				
New onset	14 (52)	9 (64)	17 (61)	8 (57)
Refractory	13 (48)	5 (36)	11 (39)	6 (43)
Disease duration (days)^c^, median (IQR)	43.0 (36.0; 273.0)	39.5 (29.0; 219.0)	41.0 (28.0; 228.5)	41.5 (34.0; 343.0)
Presence of unequivocal GCA cranial symptoms, *n* (%)	23 (85)	10 (71)	22 (79)	14 (100)
Presence of unequivocal PMR symptoms, *n* (%)	14 (52)	11 (79)	16 (57)	6 (43)
GCA diagnosis, *n* (%)				
*N*	26	13	28	14
TAB	8 (31)	2 (15)	5 (18)	8 (57)
Imaging	18 (69)	11 (85)	23 (82)	6 (43)
Imaging methodology, *n* (%)^d^				
*N*	18	11	23	6
CTA	1 (6)	0	3 (13.0)	0
MRA	1 (6)	0	1 (4)	1 (17)
PET-CT	3 (17)	7 (64)	13 (57)	4 (67)
Ultrasound	13 (72)	4 (36)	6 (26)	1 (17)
Prednisone dose at baseline^e^ (mg/day), mean (SD)	34.5 (17.0)	35.0 (14.7)	34.6 (12.9)	30.5 (15.9)
Randomization strata, *n* (%)				
< 30 mg/day prednisone^e^	12 (44)	6 (43)	12 (43)	6 (43)
≥ 30 mg/day prednisone^e^	15 (56)	8 (57)	16 (57)	8 (57)
Baseline CRP (mg/L), median (IQR)	1.6 (0.8; 3.2)	5.4 (1.1; 11.2)	2.2 (1.4; 10.6)	4.0 (2.8; 6.0)
Baseline ESR (mm/h), median (IQR)	10.0 (6.0; 28.0)	19.5 (7.0; 37.0)	17.0 (10.0; 50.0)	20.5 (12.0; 22.0)

*BMI* Body mass index, *CRP* C-reactive protein, *CTA* Computed tomography angiography, *ESR* Erythrocyte sedimentation rate, *GCA* Giant cell arteritis, *IQR* Interquartile range, *ITT* Intent-to-treat, *MRA* Magnetic resonance angiography, *n/N* Number of patients, *PBO* placebo, *PET-CT* Positron emission tomography-computed tomography, *PMR* Polymyalgia rheumatica, *SAR150/200* Sarilumab 150/200 mg, *SD* Standard deviation, *TAB* Temporal artery biopsy, *W* Week

^a^Included race not reported, other, or unknown

^b^Based on 13 patients

^c^From diagnosis date to baseline

^d^Each patient can be diagnosed by more than one imaging methodology

^e^Or prednisone equivalent dose; based on last dose prior to baseline visit

### Outcome assessments

#### Primary efficacy endpoint: achievement of sustained remission

The analysis of the primary endpoint at week 52 was limited to the week 52 analysis set and analysis of the additional endpoint of SR at week 24 was performed on the ITT population.

At week 52, nearly half of the patients in the SAR200 + 26W taper group (*n* = 6/13; 46%) and the SAR150 + 26W taper group (*n* = 3/7; 43%) achieved SR, while the proportion of patients who achieved SR was 30% (*n* = 3/10) in the PBO + 52W taper group and 0 (*n* = 0/6) in the PBO + 26W taper group (Table 2). At week 24, the proportions of patients who achieved SR in sarilumab groups were similar to those observed at week 52.

**Table 2:** Proportion of patients with SR and its components at week 52 and week 24

*n* (%)	SAR200 + 26W taper	SAR150 + 26W taper	PBO + 52W taper	PBO + 26W taper
**Primary endpoint and sensitivity analyses, *n***	13	7	10	6
Number of patients achieving SR at week 52	6 (46)	3 (43)	3 (30)	0
Number of patients achieving SR at week 52 excluding CRP	6 (46)	3 (43)	6 (60)	1 (17)
Number of patients achieving SR at week 52 excluding CRP and ESR	6 (46)	3 (43)	6 (60)	1 (17)
**Additional endpoint and sensitivity analyses, *n***	27	14	28	14
Number of patients achieving SR at week 24	13 (48)	6 (43)	11 (39)	1 (7)
Number of patients achieving SR at week 24 excluding CRP	13 (48)	6 (43)	16 (57)	5 (36)
Number of patients achieving SR at week 24 excluding CRP and ESR	13 (48)	6 (43)	16 (57)	5 (36)
**Secondary endpoints**				
** Week 52, *n***	13	7	10	6
Achievement of disease remission no later than week 12^a^	7 (54)	4 (57)	7 (70)	3 (50)
Absence of disease flare from week 12 through week 52^b^	7 (54)	4 (57)	7 (70)	3 (50)
Sustained reduction of CRP from week 12 through week 52^c^	8 (62)	5 (71)	6 (60)	3 (50)
Successful adherence to the prednisone taper from week 12 through week 52^d^	6 (46)	3 (43)	6 (60)	2 (33)
** Week 24, *n***	27	14	28	14
Achievement of disease remission no later than week 12^a^	15 (56)	9 (64)	16 (57)	6 (43)
Absence of disease flare from week 12 through week 24^b^	15 (56)	10 (71)	21 (75)	7 (50)
Sustained reduction of CRP from week 12 through week 24^c^	17 (63)	11 (79)	20 (71)	4 (29)
Successful adherence to the prednisone taper from week 12 through week 24^d^	13 (48)	7 (50)	18 (64)	5 (36)

Week 52 analysis set included patients who had an opportunity to complete the 52-week treatment period and had a randomization date prior to 16 Oct 2019. Patients who did not achieve remission, received rescue treatment with open-label prednisone (or equivalent), withdrew from the study before week 52, or had missing data that prevented assessment of the primary endpoint were considered as non-responders. Week 24 was the ITT population

*AE* Adverse event, *APR* Acute-phase reactant, *CRP* C-reactive protein, *ESR* Erythrocyte sedimentation rate, *GC* Glucocorticoid, *GCA* Giant cell arteritis, *ITT* Intent-to-treat, *n* number of patients, *PBO* Placebo, *SAR150/200* Sarilumab 150/200 mg, *SR* Sustained remission, *W* Week

^a^Disease remission is defined as resolution of signs and symptoms of GCA, and normalization of CRP (< 10 mg/L)

^b^Flare is defined as either recurrence of signs and symptoms attributable to active GCA plus an increase in GC dose due to GCA or elevation of ESR attributable to active GCA plus an increase in GC dose due to GCA

^c^The status of normalization of CRP from week 12 through week 52 was determined based on the CRP values measured at weeks 16, 20, 24, 32, 40, and 52, or at weeks 16, 20, and 24 for week 12 through week 24. If there were ≥ 2 consecutive visits with CRP ≥ 10 mg/L, then it was categorized as no normalization of CRP

^d^Successful adherence to the prednisone taper from week 12 through week 24/52 is defined as patients did not take rescue therapy from week 12 through week 24/52 and may include the use of any excess prednisone (beyond the per-protocol GC-taper regimen) with a cumulative dose of ≤ 100 mg (or equivalent), such as those employed to manage AE not related to GCA. The cumulative dose of excess prednisone use was counted from baseline to week 24/52

Results of the sensitivity analysis, which excluded CRP, showed that nearly half of the patients in the sarilumab groups (SAR200 + 26W taper: *n* = 6/13, 46%; SAR150 + 26W taper: *n* = 3/7, 43%), 60% (*n* = 6/10) in the PBO + 52W taper group, and 17% (*n* = 1/6) in the PBO + 26W taper group achieved SR at week 52. The results of the sensitivity analysis at week 24 were similar to those at week 52, except that more patients achieved SR in the PBO + 26W group (*n* = 5/14, 36%) at week 24 than week 52. The number of patients achieving SR at week 52 or week 24 did not change in the post hoc sensitivity analyses, excluding CRP and ESR (Table 2).

#### Secondary efficacy endpoints

##### Components of sustained remission:

In the week 52 analysis set, ≥ 50% of the patients in each of the four treatment groups achieved disease remission by week 12, had sustained reduction of CRP, and did not have disease flare from week 12 through week 52. The proportions of patients achieving disease remission by week 12 and those not having disease flare from week 12 through week 52 were numerically higher in the PBO + 52W taper group (*n* = 7/10, 70%) than those in the sarilumab treatment groups (SAR200 + 26W taper: *n* = 7/13, 54%; SAR150 + 26W taper: *n* = 4/7, 57%) and the PBO + 26W taper group (*n* = 3/6, 50%). At week 24, these proportions of patients were lower for the SAR200 + 26W taper group than those for the SAR150 + 26W and PBO + 52W taper groups (Table 2).

The proportions of patients who adhered to the prednisone taper from week 12 through week 52 were similar between SAR200 + 26W (*n* = 6/13, 46%) and SAR150 + 26W (*n* = 3/7, 43%) taper groups; however, the proportion was higher in the PBO + 52W taper group (*n* = 6/10, 60%). Similar trends were noted for the proportions of patients adhering to prednisone taper from week 12 through week 24 (Table 2).

Nearly half of the patients who continued the study treatment had received rescue therapy due to GCA by week 52 (SAR200 + 26W taper: *n* = 5/12, 42%; SAR150 + 26W taper: *n* = 4/7, 57%; PBO + 52W taper: *n* = 6/14, 43%; and PBO + 26W taper: *n* = 4/7, 57%) (Table 3).

**Table 3:** Number of patients who had rescue therapy due to GCA by visit—ITT population

*n* (%)	SAR200 + 26W taper	SAR150 + 26W taper	PBO + 52W taper	PBO + 26W taper
**Baseline, *n***	27	14	28	14
**Week 24**^**a**^	21	14	24	11
Had rescue therapy	7 (33)	5 (36)	5 (21)	6 (54)
Due to active GCA	7 (33)	5 (36)	4 (17)	4 (36)
Due to elevated ESR attributable to active GCA	0	0	1 (4)	2 (18)
**Week 52**^**a**^	12	7	14	7
**Had rescue therapy**	5 (42)	4 (57)	6 (43)	4 (57)
Due to active GCA	5 (42)	4 (57)	5 (36)	2 (29)
Due to elevated ESR attributable to active GCA	0	0	1 (7)	2 (29)

The number of rescue patients was calculated based on the start date and end date of rescue medication

*ESR* Erythrocyte sedimentation rate, *GCA* Giant cell arteritis, *ITT* Intent-to-treat, *n* Number of patients, *PBO* Placebo, *SAR150/200* Sarilumab 150/200 mg, *W* Week

^a^Includes patients who were in the study treatment

##### Cumulative glucocorticoid dose, giant cell arteritis flare, and glucocorticoid toxicity index:

**Fig. 3:**
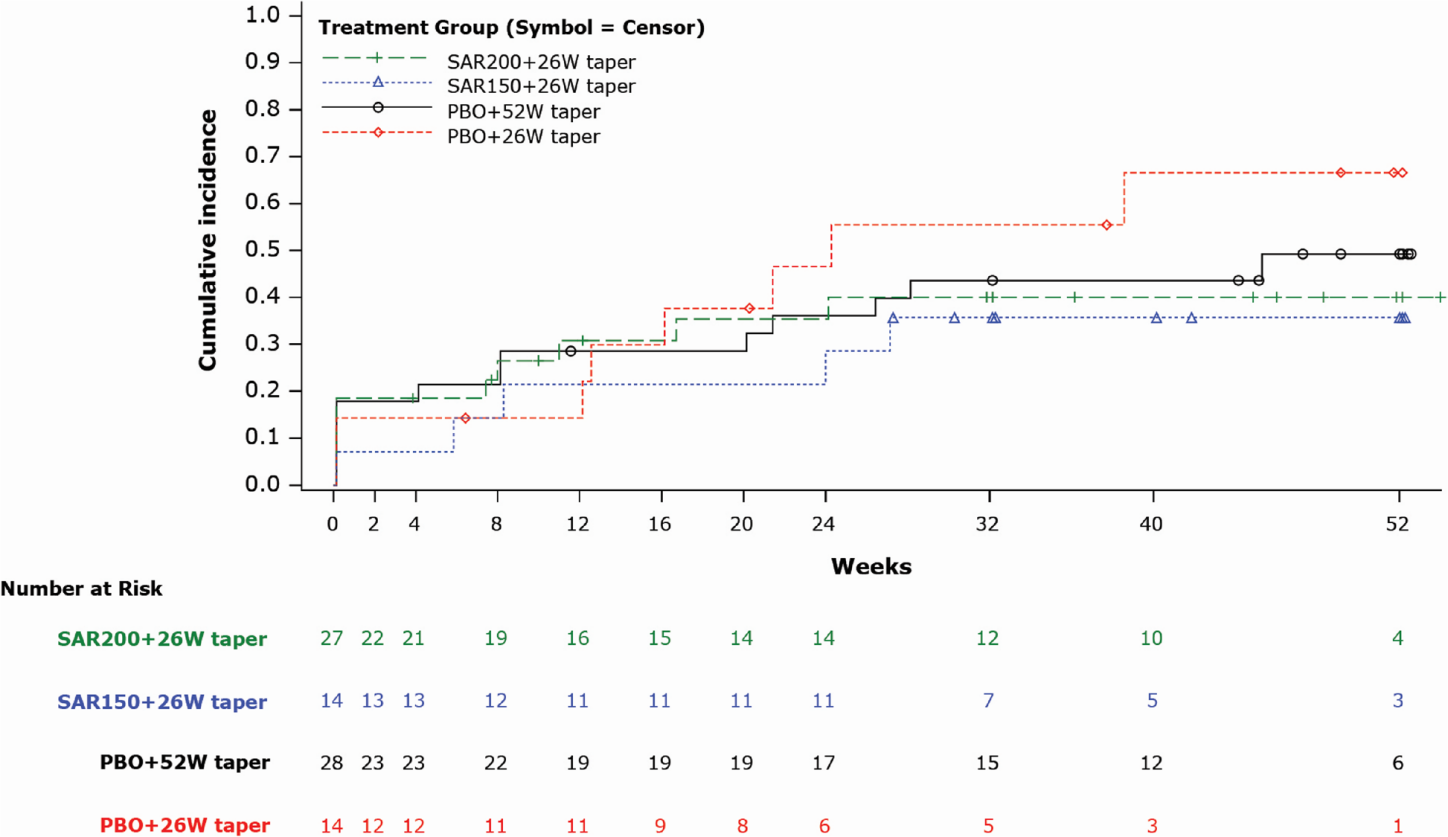
Kaplan–Meier plot: Time to first GCA flare after clinical remission until 52W (ITT population). Note: Time (days) is calculated from randomization to first GCA flare after clinical remission up to week 52. GCA, giant cell arteritis; ITT, intent-to-treat; PBO, placebo; SAR150/200, sarilumab 150/200 mg; W, week

The mean actual cumulative GC dose received by patients in the SAR200 + 26Wgroup (1643.1 mg) was lower than that received by the patients in other groups (SAR150 + 26W taper: 2177.1 mg; PBO + 52W taper: 2577.3 mg; and PBO + 26W taper: 2270.7 mg). The mean difference between the actual and the expected cumulative GC dose was highest in the PBO + 26W taper group (1010.2 mg), followed by SAR150 + 26W taper (725.1 mg), SAR200 + 26W taper (380.5 mg), and PBO + 52W taper (205.7 mg) groups (Table 4). The cumulative incidence of GCA flare remained relatively stable after week 24 in all groups, with exception of PBO + 26W taper group. Overall, the cumulative incidence of GCA flare was numerically lower in the sarilumab groups than in the PBO groups (Fig. 3).

**Table 4:** Cumulative GC dose—ITT population

Mean (SD)	SAR200 + 26W taper (*n* = 27)	SAR150 + 26W taper (*n* = 14)	PBO + 52W taper (*n* = 28)	PBO + 26W taper (*n* = 14)
Expected cumulative GC dose (mg)^a^	1262.6 (512.0)	1452.0 (464.4)	2371.6 (785.2)	1260.4 (487.1)
Actual cumulative GC dose (mg)^b^	1643.1 (967.3)	2177.1 (1326.7)	2577.3 (1018.3)	2270.7 (1418.0)
Difference between actual and expected cumulative GC dose (mg)	380.5 (761.7)	725.1 (1185.4)	205.7 (742.8)	1010.2 (1336.8)

*GC* Glucocorticoid, *GCA *Giant cell arteritis, *ITT *Intent-to-treat, *n *Number of patients, *PBO *Placebo, *SAR150/200 *Sarilumab 150/200 mg, *SD *Standard deviation, *W *Week

^a^Expected cumulative dose based on the GC-taper regimen up to end of treatment, assuming that the taper was continued without error

^b^Cumulative dose of GC used for GCA disease is up to the end of treatment, including expected prednisone in taper regimen per protocol, GC used in rescue therapy, and use of commercial prednisone

The detailed results of GTI scores are presented in Table 5. The mean CWS of GTI showed a trend for lower toxicity in the SAR200 + 26W taper group at week 52 (52.8 [39.0] in the SAR200 + 26W taper group; 77.2 [41.7] in the SAR150 + 26W taper group, 73.0 [50.3] in the PBO + 52W taper group, and 84.7 [33.4] in the PBO + 26W group), although there was no difference noted between SAR200 + 26W taper and PBO groups at week 24. The mean AIS showed a trend for improvement in the SAR200 + 26W taper group and the PBO + 52W taper group at week 52 (− 0.5 [51.5] in the SAR200 + 26W taper group; 23.7 [31.9] in the SAR150 + 26W taper group; − 19.5 [65.0] in the PBO + 52W taper group; and 31.2 [54.7] in the PBO + 26W group). At week 24, the AIS scores in both PBO groups and SAR200 + 26W taper group were found to be improving.

**Table 5:** GTI scores^a^—week 52 analysis set and ITT population

GTI components^b^	SAR200 + 26W taper	SAR150 + 26W taper	PBO + 52W taper	PBO + 26W taper
**GTI components at week 52**				
Number at baseline, *n*	13	7	10	6
Ongoing at week 52, *n* (%)	8 (62)	6 (86)	9 (90)	6 (100)
Missing and imputed, *n* (%)	1 (8)	0	3 (30)	1 (17)
*CWS*				
Mean (SD)	52.8 (39.0)	77.2 (41.7)	73.0 (50.3)	84.7 (33.4)
Min; max	10; 150	20; 127	0; 145	51; 141
*AIS*				
Mean (SD)	-0.5 (51.5)	23.7 (31.9)	-19.5 (65.0)	31.2 (54.7)
Min; max	-142; 40	-11; 69	-157; 49	-54; 90
**GTI components at week 24**				
Number at baseline, *n*	27	14	28	14
Ongoing at week 24, *n* (%)	18 (67)	13 (93)	24 (86)	9 (64)
Missing and imputed, *n* (%)	1 (4)	2 (14)	3 (11)	2 (14)
*CWS*				
Mean (SD)	31.0 (42.9)	55.1 (43.1)	29.2 (30.8)	30.7 (33.2)
Min; max	0; 196	10; 166	0; 115	0; 103
*AIS*				
Mean (SD)	-3.3 (43.4)	14.2 (55.0)	-21.6 (54.8)	-13.4 (44.3)
Min; max	-109; 112	-43; 166	-156; 74	-115; 65

*AIS *Aggregate improvement score, *CWS *Cumulative worsening score, *GC *Glucocorticoid, *GTI *Glucocorticoid toxicity index, *ITT *Intent-to-treat, *n *Number of patients, *PBO *Placebo, *SAR150/200 *Sarilumab 150/200 mg, *SD *Standard deviation, *W *Week

^a^Smaller GTI score implies less GC toxicity

^b^Score ranges from − 36 to 439, based on the information from clinical laboratory assessments, vital sign assessments, concomitant medications, and clinical assessments

#### Safety

The cumulative exposure to study drug was 17.4 patient-years in the SAR200 + 26W taper group, 10.8 patient-years in the SAR150 + 26W taper group, 21.2 patient-years in the PBO + 52W taper group, and 9.4 patient-years in the PBO + 26W taper group; the majority of patients in all groups were treated for > 40 and < 52 weeks.

Most patients (80–100%) in all treatment groups experienced treatment-emergent adverse events (TEAEs) (Additional file 4; Table S4). The most common TEAEs by preferred term (PT) (reported in > 15% patients in any treatment group) were mania, injection site reaction, arthralgia, diarrhea, cognitive disorder, headache, depression, and increased tendency to bruise.

The proportion of patients with serious adverse events (SAEs) was highest in the SAR200 + 26W taper group (*n* = 7/27, 26%), followed by PBO + 26W taper group (*n* = 3/14, 21%), SAR150 + 26W taper group (*n* = 2/14, 14%), and PBO + 52W taper group (*n* = 2/28, 7%). In each group, all SAEs were reported by one patient, except for neutropenia, which was reported by two patients in the SAR200 + 26W taper group. Serious infections were reported in two patients in the SAR200 + 26W taper group (COVID-19, cellulitis, and urosepsis), and in one patient each in the PBO + 52W taper group (lower respiratory tract infection) and the PBO + 26W taper group (pyelonephritis and septic shock). One patient in the SAR150 + 26W taper group had SAEs of retinal artery occlusion and unilateral blindness on day 57 of the study. The patient saw an eye specialist, who did not feel that GCA was responsible for the visual disturbance. The investigator’s assessment was “caused by arteriosclerotic reasons,” and that both SAEs were not related to GCA. However, a correlation cannot be completely ruled out. Another patient in the SAR200 + 26W taper group was diagnosed with an SAE of aortic dissection on day 233 of the study, detected during an unrelated magnetic resonance imaging, and confirmed with a computed tomography scan. The patient was asymptomatic, and the SAE was unrelated to the study drug as assessed by the investigator (Table 6).

**Table 6:** Safety summary

PT, *n* (%)^a^	SAR200 + 26W taper (*n* = 27)	SAR150 + 26W taper (*n* = 14)	PBO + 52W taper (*n* = 28)	PBO + 26W taper (*n* = 14)
**Treatment-emergent SAE**	**7 (26)**	**2 (14)**	**2 (7)**	**3 (21)**
COVID-19	1 (4)	0	0	0
Cellulitis	1 (4)	0	0	0
Urosepsis	1 (4)	0	0	0
Lower respiratory tract infection	0	0	1 (4)	0
Pyelonephritis	0	0	0	1 (7)
Septic shock	0	0	0	1 (7)
Neutropenia	2 (7)	0	0	0
Cerebral amyloid angiopathy	1 (4)	0	0	0
Blindness unilateral	0	1 (7)	0	0
Retinal artery occlusion	0	1 (7)	0	0
Aortic dissection	1 (4)	0	0	0
Deep vein thrombosis	0	0	1 (4)	0
Peripheral vascular disorder	0	0	0	1 (7)
Acute respiratory failure	0	0	0	1 (7)
Colitis ulcerative	0	0	1 (4)	0
Hiatus hernia	0	1 (7)	0	0
Femur fracture	1 (4)	0	0	0
**TEAE leading to permanent treatment discontinuation**	**7 (26)**	**1 (7)**	**2 (7)**	**1 (7)**
COVID-19	1 (4)	0	0	0
Cellulitis	1 (4)	0	0	0
Ovarian adenoma	0	1 (7)	0	0
Neutropenia	2 (7)	0	0	0
Thyroid cyst	0	0	1 (4)	0
Cerebral amyloid angiopathy	1 (4)	0	0	0
Headache	1 (4)	0	0	0
Atrial fibrillation	1 (4)	0	0	0
Acute respiratory failure	0	0	0	1 (7)
Gastrointestinal disorder	1 (4)	0	0	0
Colitis ulcerative	0	0	1 (4)	0
Alanine aminotransferase increased	1 (4)	0	0	0
**AESI**	**6 (22)**	**1 (7)**	**2 (7)**	**1 (7)**
Herpes ophthalmic	1 (4)	0	0	0
Herpes zoster^b^	0	1 (7)	1 (4)	0
Cellulitis	1 (4)	0	0	0
COVID-19	1 (4)	0	0	0
Cellulitis	1 (4)	0	0	0
Herpes zoster^c^	0	0	1 (4)	0
Lower respiratory tract infection	0	0	1 (4)	0
Pyelonephritis	0	0	0	1 (7)
Septic shock	0	0	0	1 (7)
ALT increased^d^	1 (4)	0	0	0
Neutropenia	2 (7)	0	0	0
ALT increased^e^	1 (4)	0	0	0

*AESI* Adverse event of special interest, *ALT* Alanine aminotransferase, *COVID-19* Coronavirus disease-2019, *MedDRA* Medical Dictionary for Regulatory Activities, *PBO* Placebo, *PT* Preferred term, *SAR150/200* Sarilumab 150/200 mg, *SAE* Serious adverse event, *TEAE* Treatment-emergent adverse event, *W* Week

^a^MedDRA 23.1; *n* (%) = number and percentage of patients with at least one SAE/TEAE/AESI

^b^Opportunistic infection

^c^Infection requiring parenteral treatment

^d^ALT increase leading to permanent discontinuation

^e^ALT increase ≥ 3 upper limit of normal

The TEAEs leading to treatment discontinuation were reported in 26% patients in the SAR200 + 26W taper group (*n* = 7/27) and in 7% patients each in the SAR150 + 26W taper group (*n* = 1/14), PBO + 52W taper group (*n* = 2/28), and PBO + 26W taper group (*n* = 1/14) (Table 6).

The proportion of patients with at least one treatment-emergent adverse event of special interest (AESI; 22%) and the number of reported AESIs (*n* = 6/27) were highest in the SAR200 + 26W taper group (Table 6).

There were three deaths reported during the study: one in the PBO + 26W taper group due to acute respiratory failure and two in the SAR200 + 26W taper group due to urosepsis and COVID-19. The death due to urosepsis occurred 33 days after the last dose of sarilumab had been administered. It was assessed by the study investigator as probably related to the study drug, although this patient had a complicated disease course, and the event was not associated with neutropenia. The death due to COVID-19 occurred 26 days after the last dose of sarilumab and was assessed by the investigator as not related to the study drug.

The mean neutrophil count declined after week 12 in all treatment groups, with a higher proportion of patients with neutropenia in sarilumab groups (SAR200 + 26W taper: *n* = 7/25, 28%; SAR150 + 26W taper: *n* = 4/14, 28%) compared with PBO groups (PBO + 52W taper: *n* = 1/27, 4%; PBO + 26W taper: *n* = 0/13). The cumulative incidence of Grade 3 or 4 neutropenia was higher in the sarilumab groups than that in the placebo groups and remained stable from week 8 through week 52 (Additional file 5; Fig. S1). There were no significant changes in the platelet count in all treatment groups; only one (7%) patient in the SAR150 + 26W taper group had Grade 1 thrombocytopenia after week 24. In the SAR200 + 26W taper group, one patient (4%) experienced an increased alanine aminotransferase of ≥ 3 ULN.

#### Pharmacodynamics

From week 12 through week 52, CRP levels in the sarilumab groups were maintained at < 10 mg/L and were lower than that observed in the PBO groups (Additional file 6; Fig. S2).

**Fig. 4:**
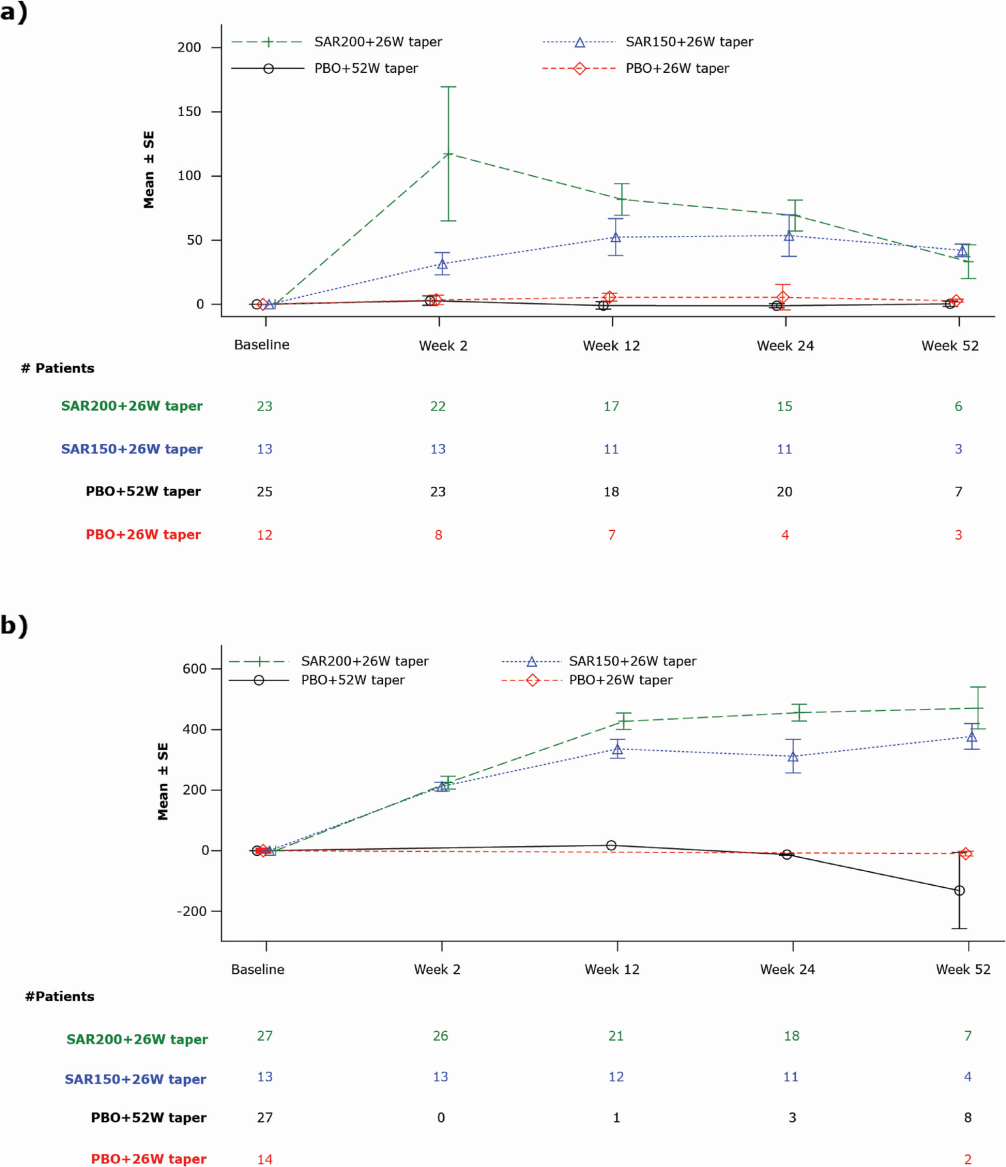
Change from baseline in IL-6 and sIL-6R levels during the 52W treatment period — safety population: **a**) mean change in IL-6 levels (ng/L) over time and **b**) mean change in sIL-6R levels (ng/mL) over time. IL-6, interleukin-6; PBO, placebo; Q2W, every 2 weeks; SAR150/200, sarilumab 150/200 mg; SE, standard error; sIL-6R, soluble IL-6 receptor; W, week

Mean changes in IL-6 and sIL-6R levels over time from baseline through week 52 are depicted in Fig. 4. Overall, IL-6 levels increased transiently in sarilumab groups, followed by a decline and maintenance at low levels; the increase in IL-6 levels was greater and rapid at the higher dose of 200 mg. No changes were observed in the levels of IL-6 in the placebo groups. This was consistent with the changes noted in the levels of sIL-6R, which increased over time in sarilumab groups, with no changes noted in the placebo groups.

#### Pharmacokinetics

The PK analysis set comprised 26 patients in the SAR200 + 26W taper group and 14 patients in the SAR150 + 26W taper group. All pre-dose concentrations of functional sarilumab in serum at week 0 were below the lower limit of quantification (312.5 ng/mL). After multiple SC administrations of sarilumab, the observed trough concentrations of functional sarilumab increased over time from week 0 through week 52 in sarilumab groups. At week 24, the mean trough concentration of functional sarilumab increased 2.7-fold with a 1.3-fold increase in dose. There was an accumulation of sarilumab following SC administrations of sarilumab 150 and 200 mg, with the accumulation ratio of ~ 9-fold based on the mean trough concentrations (Additional file 7; Fig. S3).

## Discussion

This study was designed to evaluate the efficacy and safety of sarilumab in patients with GCA; however, it was prematurely terminated, resulting in low enrollment and a limited dataset for statistical analyses. Due to the decision to discontinue all patients from the study, the interpretation of the study results was severely impeded. The decision to prematurely terminate the study was not driven by any safety issues with the administration of sarilumab.

In this study, both CRP and ESR were used as a part of inclusion criteria as these are the usual inflammatory markers that correlate with disease activity and are used in general clinical practice for the monitoring of patients’ disease activity. Further, higher historical cutoff values were used for these inflammatory markers (CRP > 25 mg/L or ESR ≥ 50 mm/h) as per the study protocol eligibility criteria, to ensure a greater likelihood that the patients enrolled in the study truly had active GCA. Similar CRP and ESR levels have been required in previous trials investigating other IL-6R inhibitors for including patients [17, 26].

In this study, almost half of the patients in the SAR200 + 26W and SAR150 + 26W taper groups achieved SR at week 52 and week 24, which was numerically higher than that observed in the placebo groups; CRP was included as a part of the definition for disease remission, along with its normalization value of < 10 mg/L, consistent with the GiACTA trial [16]. The higher rate of SR in sarilumab than placebo groups may have been driven by the resolution of CRP, as evident through the sensitivity analysis excluding APR. The sensitivity analyses (excluding APR from the definition of SR) showed a greater number of patients achieving SR in the PBO + 52W taper group than in the sarilumab groups.

Unlike the GiACTA study of TCZ [16], the present study was conducted in the context of an approved IL-6Ri treatment for GCA being available (i.e., TCZ). Therefore, the investigators might have been less willing to recruit their most severe GCA patients to the present trial, compared with the situation at the time of GiACTA recruitment. Further, compared with GiACTA, this study enrolled a greater number of newly diagnosed GCA patients (52–64% vs 45–52%), who were less likely to relapse. This might have partially explained the 30% SR for the PBO + 52W taper group in this study (higher than the 18% reported in GiACTA) [16].

In this study, there was some suggestion of a GC-sparing effect of 200 mg sarilumab, as shown by the reduced cumulative GC dose in the SAR200 + 26W taper group, but this result was not consistently observed for the lower sarilumab dose (150 mg).

A majority of patients in all treatment groups experienced TEAEs, with neutropenia more frequently reported in patients who received sarilumab, as expected due to the known PD effect of IL-6R inhibition on neutrophil counts. One patient had SAEs of retinal artery occlusion and unilateral blindness, which were deemed by the investigator to be unrelated to the study drug. Overall, sarilumab was tolerable with a safety profile consistent with the previous studies in RA [27] and IL-6 receptor inhibition [28–30].

Although the CRP levels varied among the study groups, they were maintained at < 10 mg/L in the sarilumab groups and were lower than those observed in the placebo groups. This aligns with the previously published data of sarilumab in patients with RA, and attributes to the blockade of IL-6 signaling pathway and subsequent inhibition of the inflammatory response by sarilumab [31, 32]. Overall, the PK/PD profile of sarilumab was consistent with the previous studies, showing increased concentrations of sarilumab and IL-6 over time, along with an increased receptor occupancy [22, 33–35].

The main limitation of the study was its small sample size due to the early termination of the study with protracted recruitment timelines, exacerbated by the COVID-19 pandemic.

## Conclusions

The planned recruitment of this study was not achieved, as it was stopped early by the sponsor due to slow recruitment and the COVID-19 pandemic. Therefore, it is difficult to draw clear conclusions regarding the efficacy of sarilumab in GCA.

### Supplementary Information

**Additional file 1:** **Table S1. **Inclusion and exclusion criteria.

**Additional file 2: Table S2. **Standardized 26-week GC-taper regimen during the study treatment period.

**Additional file 3:** **Table S3. **Standardized 52-week GC-taper regimen during the study treatment period.

**Additional file 4:** **Table S4. **Number (%) of patients experiencing TEAE(s) by primary SOC – safety population.

**Additional file 5:** **Fig. S1. **Kaplan–Meier plot for time to onset of the initial Grade 3 or Grade 4 neutropenia (neutrophil count<1.0 G/L).

**Additional file 6:** **Fig. S2. **Mean CRP at each visit during the TEAE period – safety population.

**Additional file 7:** **Fig. S3. **Serum sarilumab trough concentrations during the TEAE period – PK population: a) Functional mean (SD) concentration over time; b) Functional geometric mean concentration over time.

## Data Availability

Qualified researchers may request access to patient-level data and related documents (including, e.g., the clinical study report, study protocol with any amendments, blank case report form, statistical analysis plan, and dataset specifications). Patient-level data will be anonymized, and study documents will be redacted to protect the privacy of trial participants. Further details on Sanofi’s data sharing criteria, eligible studies, and process for requesting access can be found at https://vivli.org/.

## Abbreviations

AE: Adverse event
AESI: Adverse event of special interest
AIS: Aggregate improvement score
ALT: Alanine aminotransferase
BMI: Body mass index
COVID-19: Coronavirus disease-2019
CRP: C-reactive protein
CS: Corticosteroid
CTA: Computed tomography angiography
CWS: Cumulative worsening score
D: Day
EOS: End of study
EOT: End of treatment
ESR: Erythrocyte sedimentation rate
GC: Glucocorticoid
GCA: Giant cell arteritis
GTI: Glucocorticoid toxicity index
IEC: Independent ethics committee
IL-6: Interleukin-6
IL-6Ri: Interleukin-6-receptor inhibitor
IQR: Interquartile range
IRB: Institutional review board
ITT: Intent-to-treat
IV: Intravenous
MedDRA: Medical Dictionary for Regulatory Activities
MRA: Magnetic resonance angiography
*n*/*N*: Number of patients
NC: Not calculated
PBO: Placebo
PD: Pharmacodynamics
PET-CT: Positron emission tomography-computed tomography
PK: Pharmacokinetics
PMR: Polymyalgia rheumatica
PS: Prednisone
PT: Preferred term
Q2W: Every 2 weeks
R: Randomization
RA: Rheumatoid arthritis
SAE: Serious adverse event
SAR150: Sarilumab 150 mg
SAR200: Sarilumab 200 mg
SC: Subcutaneous
SD: Standard deviation
SE: Standard error
sIL-6R: Soluble interleukin-6 receptor
SOC: System organ class
SR: Sustained remission
TAB: Temporal artery biopsy
TB: Tuberculosis
TCZ: Tocilizumab
TEAE: Treatment-emergent adverse event
ULN: Upper limit of normal
V: Visit
W: Week
