# Supplementary material for: A phase 3 randomized, double-blind, placebo-controlled study to evaluate the efficacy and safety of sarilumab in patients with giant cell arteritis
Source: Arthritis Res Ther. 2023 Oct 16;25:199. doi: 10.1186/s13075-023-03177-6 (PMC10577982; doi:10.1186/s13075-023-03177-6)
Supplement: Supplementary file 1 — Additional file 1: Table S1. Inclusion and exclusion criteria. [file 13075_2023_3177_MOESM1_ESM.docx]

**Additional file 1**

**Table S1** Inclusion and exclusion criteria

| **Inclusion criteria:** |
| --- |
| - Diagnosis of GCA according to the following criteria:   - ≥50 years of age   - History of ESR ≥50 mm/h (or CRP >25 mg/L)   - Unequivocal cranial symptoms of GCA or PMR   - Presence of at least one of the following: TAB revealing features of GCA and evidence of large-vessel vasculitis by angiography or cross-sectional imaging (angiography, CTA, MRA or PET-CT, and ultrasound) - New-onset active disease (diagnosis within 6W of baseline) or refractory active GCA (diagnosis >6 weeks before baseline and previous treatment with ≥40 mg/day prednisone [or equivalent] for at least consecutive 2 weeks at a time) - Symptoms of GCA within 6 weeks of baseline including unequivocal cranial symptoms of GCA or PMR, or other features judged by the investigator to be consistent with GCA or PMR flares - Either ESR ≥30 mm/h or CRP ≥10 mg/L within 6 weeks of baseline - Receiving or able to receive prednisone 20–60 mg/day for the treatment of active GCA |
| **Exclusion criteria:** |
| - Organ transplantation recipients (except corneas, unless it was within three months prior to the baseline visit) - Major ischemic event (unrelated to GCA) within 12 weeks of screening - Prior treatment of GCA with any of the following:   - Janus kinase inhibitor within 4 weeks of baseline   - Cell-depletion agents without evidence of recovery of B cells to baseline level   - Tumor necrosis factor inhibitors within 2–8 weeks or less than at least 5 half-lives have elapsed prior to baseline, whichever is longer   - Abatacept within 8 weeks of baseline   - Anakinra within 1 week of baseline - Use of alkylating agents including cyclophosphamide within 6 months of baseline - Use of immunosuppressants within 4 weeks of baseline (use of methotrexate <25 mg/week that had been stable for at least 3 months prior to baseline was not exclusionary) - Therapeutic failure with a biological IL-6R antagonist - Concurrent use of systemic CS for conditions other than GCA, or use of IV CS at a dose equivalent to ≥100 mg of methylprednisolone within 8 weeks of baseline for GCA therapy - History of alcohol or drug abuse within 5 years prior to the screening visit - History of invasive opportunistic infections, recurrent herpes zoster or active herpes zoster, prior articular or prosthetic joint infection, active TB, or prior incompletely treated TB - Patients who received any live, attenuated vaccine within 3 months prior to the baseline visit - Patients who had a positive test at screening for hepatitis B, hepatitis C, or human immunodeficiency virus - Evidence of serious, uncontrolled concomitant disease (e.g., cardiovascular, respiratory, hepatic, renal, endocrine etc.) - Past or current history of malignancy, other than adequately treated carcinoma *in situ* of the cervix, non-metastatic squamous cell or basal cell carcinoma of the skin, within 5 years prior to the baseline visit - Patients with a history of inflammatory bowel disease, or severe diverticulitis, or previous gastrointestinal perforation |
| CRP, C-reactive protein; CS, corticosteroid; CTA, computed tomography angiography; ESR, erythrocyte sedimentation rate; GCA, giant cell arteritis; IL-6R, interleukin-6-receptor; IV, intravenous; MRA, magnetic resonance angiography; PET-CT, positron emission tomography-computed tomography; PMR, polymyalgia rheumatica; TAB, temporal artery biopsy; TB, tuberculosis; W, week |
