# Supplementary material for: A phase 3 randomized, double-blind, placebo-controlled study to evaluate the efficacy and safety of sarilumab in patients with giant cell arteritis
Source: Arthritis Res Ther. 2023 Oct 16;25:199. doi: 10.1186/s13075-023-03177-6 (PMC10577982; doi:10.1186/s13075-023-03177-6)
Supplement: Supplementary file 2 — Additional file 2: Table S2. Standardized 26-week GC-taper regimen during the study treatment period. [file 13075_2023_3177_MOESM2_ESM.docx]

**Additional file 2**

**Table S2** Standardized 26-week GC-taper regimen during the study treatment period

| **Week** | **Daily PS dose 26-week taper regimen (mg/day)** | | | | | | |
| --- | --- | --- | --- | --- | --- | --- | --- |
| 0 (Day 1)  *PS starting dose* | 60^a^ | 50^a^ | 40^a^ | 35^a^ | 30^a^ | 25^a^ | 20^a^ |
| 1 | 50 ^a^ | 40^a^ | 35^a^ | 30^a^ | 25^a^ | 20^a^ | 15 |
| 2 | 40^a^ | 35^a^ | 30^a^ | 25^a^ | 20^a^ | 15 | 13 |
| 3 | 35^a^ | 30^a^ | 25^a^ | 20^a^ | 15 | 13 | 12 |
| 4 | 30^a^ | 25^a^ | 20^a^ | 15 | 13 | 12 | 10 |
| 5 | 25^a^ | 20^a^ | 15 | 13 | 12 | 10 | 9 |
| 6 | 20^a^ | 15 | 13 | 12 | 10 | 9 | 8 |
| 7 | 15 | 13 | 12 | 10 | 9 | 8 | 7 |
| 8 | 13 | 12 | 10 | 9 | 8 | 7 | 6 |
| 9 | 12 | 10 | 9 | 8 | 7 | 6 | 6 |
| 10 | 10 | 9 | 8 | 7 | 6 | 6 | 5 |
| 11 | 9 | 8 | 7 | 6 | 6 | 5 | 5 |
| 12 | 8 | 7 | 6 | 6 | 5 | 5 | 4 |
| 13 | 7 | 6 | 6 | 5 | 5 | 4 | 4 |
| 14 | 6 | 6 | 5 | 5 | 4 | 4 | 3 |
| 15 | 6 | 5 | 5 | 4 | 4 | 3 | 3 |
| 16 | 5 | 5 | 4 | 4 | 3 | 3 | 2 |
| 17 | 5 | 4 | 4 | 3 | 3 | 2 | 2 |
| 18 | 4 | 4 | 3 | 3 | 2 | 2 | 1 |
| 19 | 4 | 3 | 3 | 2 | 2 | 1 | 1 |
| 20 | 3 | 3 | 2 | 2 | 1 | 1 | PS PBO^b^ |
| 21 | 3 | 2 | 2 | 1 | 1 | PS PBO^b^ | PS PBO^b^ |
| 22 | 2 | 2 | 1 | 1 | PS PBO^b^ | PS PBO^b^ | PS PBO^b^ |
| 23 | 2 | 1 | 1 | PS PBO^b^ | PS PBO^b^ | PS PBO^b^ | PS PBO^b^ |
| 24 | 1 | 1 | PS PBO^b^ | PS PBO^b^ | PS PBO^b^ | PS PBO^b^ | PS PBO^b^ |
| 25 | 1 | PS PBO^b^ | PS PBO^b^ | PS PBO^b^ | PS PBO^b^ | PS PBO^b^ | PS PBO^b^ |
| 26–45 | PS PBO^b^ | PS PBO^b^ | PS PBO^b^ | PS PBO^b^ | PS PBO^b^ | PS PBO^b^ | PS PBO^b^ |
| 46 | PS PBO^b^ | PS PBO^b^ | PS PBO^b^ | PS PBO^b^ | PS PBO^b^ | PS PBO^b^ | No PS dosing |
| 47 | PS PBO^b^ | PS PBO^b^ | PS PBO^b^ | PS PBO^b^ | PS PBO^b^ | No PS dosing | No PS dosing |
| 48 | PS PBO^b^ | PS PBO^b^ | PS PBO^b^ | PS PBO^b^ | No PS dosing | No PS dosing | No PS dosing |
| 49 | PS PBO^b^ | PS PBO^b^ | PS PBO^b^ | No PS dosing | No PS dosing | No PS dosing | No PS dosing |
| 50 | PS PBO^b^ | PS PBO^b^ | No PS dosing | No PS dosing | No PS dosing | No PS dosing | No PS dosing |
| 51 | PS PBO^b^ | No PS dosing | No PS dosing | No PS dosing | No PS dosing | No PS dosing | No PS dosing |
| ^a^PS was provided as open-label.  ^b^PS matching placebo.  GC, glucocorticoid; PBO, placebo; PS, prednisone | | | | | | | |
