# Supplementary material for: A phase 3 randomized, double-blind, placebo-controlled study to evaluate the efficacy and safety of sarilumab in patients with giant cell arteritis
Source: Arthritis Res Ther. 2023 Oct 16;25:199. doi: 10.1186/s13075-023-03177-6 (PMC10577982; doi:10.1186/s13075-023-03177-6)
Supplement: Supplementary file 4 — Additional file 4: Table S4. Number (%) of patients experiencing TEAE(s) by primary SOC – safety population. [file 13075_2023_3177_MOESM4_ESM.docx]

**Additional file 4**

**Table S4** Number (%) of patients experiencing TEAE(s) by primary SOC – safety population

| **Safety population (*N* = 83)^a^** | **SAR200+26W taper (*n* = 27)** | **SAR150+26W taper (*n* = 14)** | **PBO+52W taper (*n* = 28)** | **PBO+26W taper (*n* = 14)** |
| --- | --- | --- | --- | --- |
| **Any class** | 22 (82) | 13 (93) | 24 (86) | 14 (100) |
| Infections and infestations | 11 (41) | 4 (29) | 9 (32) | 5 (36) |
| Neoplasms benign, malignant, and unspecified (including cysts and polyps) | 1 (4) | 1 (7) | 1 (4) | 0 |
| Blood and lymphatic system disorders | 6 (22) | 5 (36) | 5 (18) | 3 (21) |
| Immune system disorders | 1 (4) | 0 | 0 | 0 |
| Endocrine disorders | 1 (4) | 0 | 2 (7) | 2 (14) |
| Metabolism and nutrition disorders | 2 (7) | 2 (14) | 3 (11) | 2 (14) |
| Psychiatric disorders | 3 (11) | 2 (14) | 8 (29) | 7 (50) |
| Nervous system disorders | 6 (22) | 4 (29) | 6 (21) | 6 (43) |
| Eye disorders | 5 (19) | 3 (21) | 2 (7) | 5 (36) |
| Ear and labyrinth disorders | 1 (4) | 0 | 0 | 3 (21) |
| Cardiac disorders | 1 (4) | 0 | 2 (7) | 3 (21) |
| Vascular disorders | 5 (19) | 3 (21) | 3 (11) | 3 (21) |
| Respiratory, thoracic, and mediastinal disorders | 4 (15) | 3 (21) | 4 (14) | 5 (36) |
| Gastrointestinal disorders | 11 (41) | 4 (29) | 9 (32) | 6 (43) |
| Skin and subcutaneous tissue disorders | 7 (26) | 6 (43) | 7 (25) | 8 (57) |
| Musculoskeletal and connective tissue disorders | 6 (22) | 8 (57) | 7 (25) | 5 (36) |
| Renal and urinary disorders | 1 (4) | 0 | 1 (4) | 0 |
| Reproductive system and breast disorders | 0 | 0 | 0 | 1 (7) |
| General disorders and administration site conditions | 6 (22) | 5 (36) | 5 (18) | 3 (21) |
| Investigations | 8 (30) | 0 | 1 (4) | 2 (14) |
| Injury, poisoning, and procedural complications | 8 (30) | 2 (14) | 2 (7) | 4 (29) |
| ^a^MedDRA 23.1, *n* (%) = number and percentage of patients with at least one TEAE.  MedDRA, Medical Dictionary for Regulatory Activities; PBO, placebo; SAR150/200, sarilumab 150/200 mg; SOC, system organ class; TEAE: treatment-emergent adverse event; W, week | | | | |
