# Supplementary material for: A phase 3 randomized, double-blind, placebo-controlled study to evaluate the efficacy and safety of sarilumab in patients with giant cell arteritis
Source: Arthritis Res Ther. 2023 Oct 16;25:199. doi: 10.1186/s13075-023-03177-6 (PMC10577982; doi:10.1186/s13075-023-03177-6)
Supplement: Supplementary file 5 — Additional file 5: Fig. S1. Kaplan–Meier plot for time to onset of the initial Grade 3 or Grade 4 neutropenia (neutrophil count<1.0 G/L). [file 13075_2023_3177_MOESM5_ESM.docx]

**Additional file 5**

**Fig. S1** Kaplan–Meier plot for time to onset of the initial Grade 3 or Grade 4 neutropenia (neutrophil count <1.0 G/L)


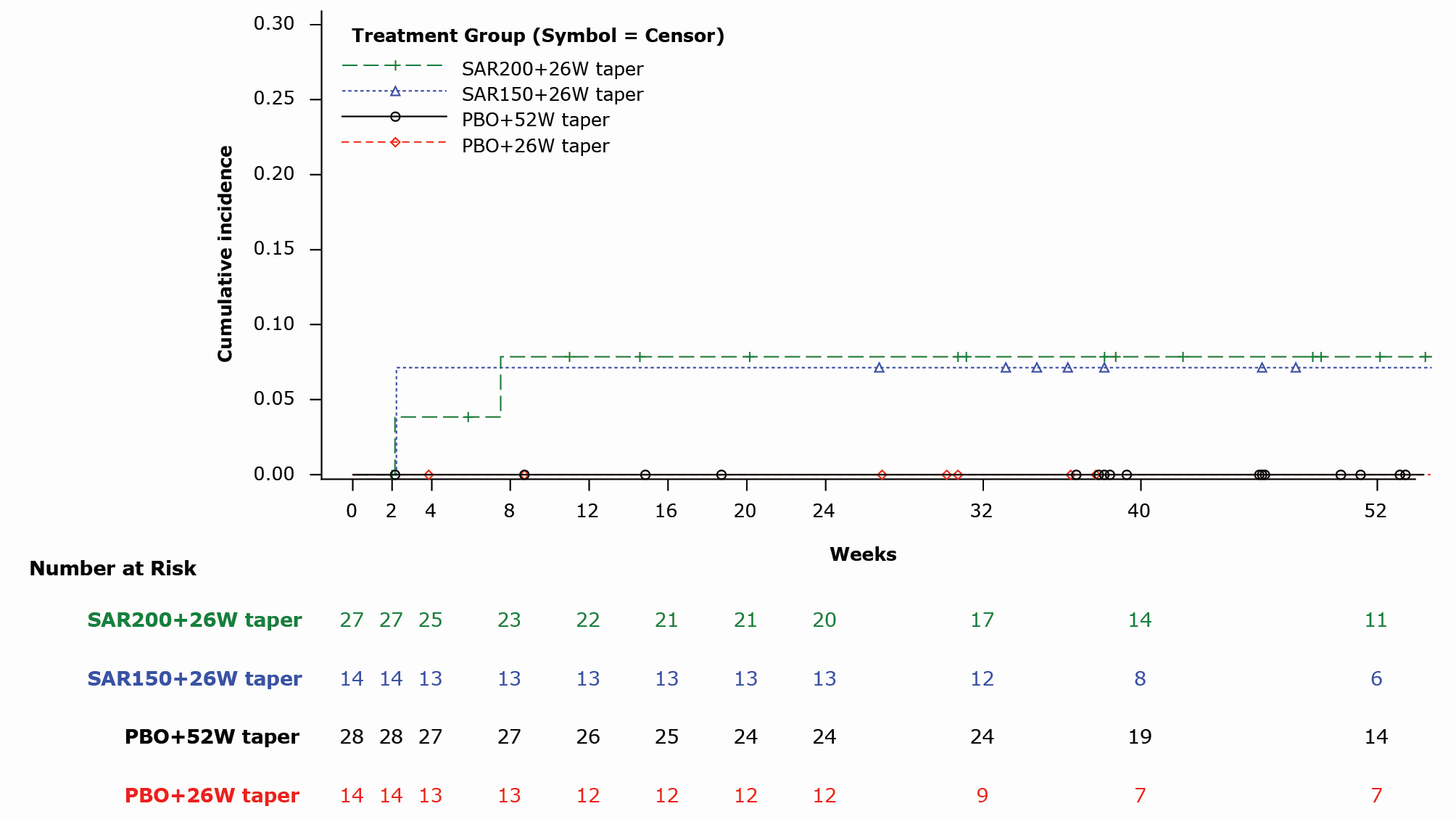


PBO, placebo; SAR150/200, sarilumab 150/200 mg; W, week
