# Supplementary material for: A phase 3 randomized, double-blind, placebo-controlled study to evaluate the efficacy and safety of sarilumab in patients with giant cell arteritis
Source: Arthritis Res Ther. 2023 Oct 16;25:199. doi: 10.1186/s13075-023-03177-6 (PMC10577982; doi:10.1186/s13075-023-03177-6)
Supplement: Supplementary file 6 — Additional file 6: Fig. S2. Mean CRP at each visit during the TEAE period – safety population. [file 13075_2023_3177_MOESM6_ESM.docx]

**Additional file 6**

**Fig. S2** Mean CRP at each visit during the TEAE period – safety population


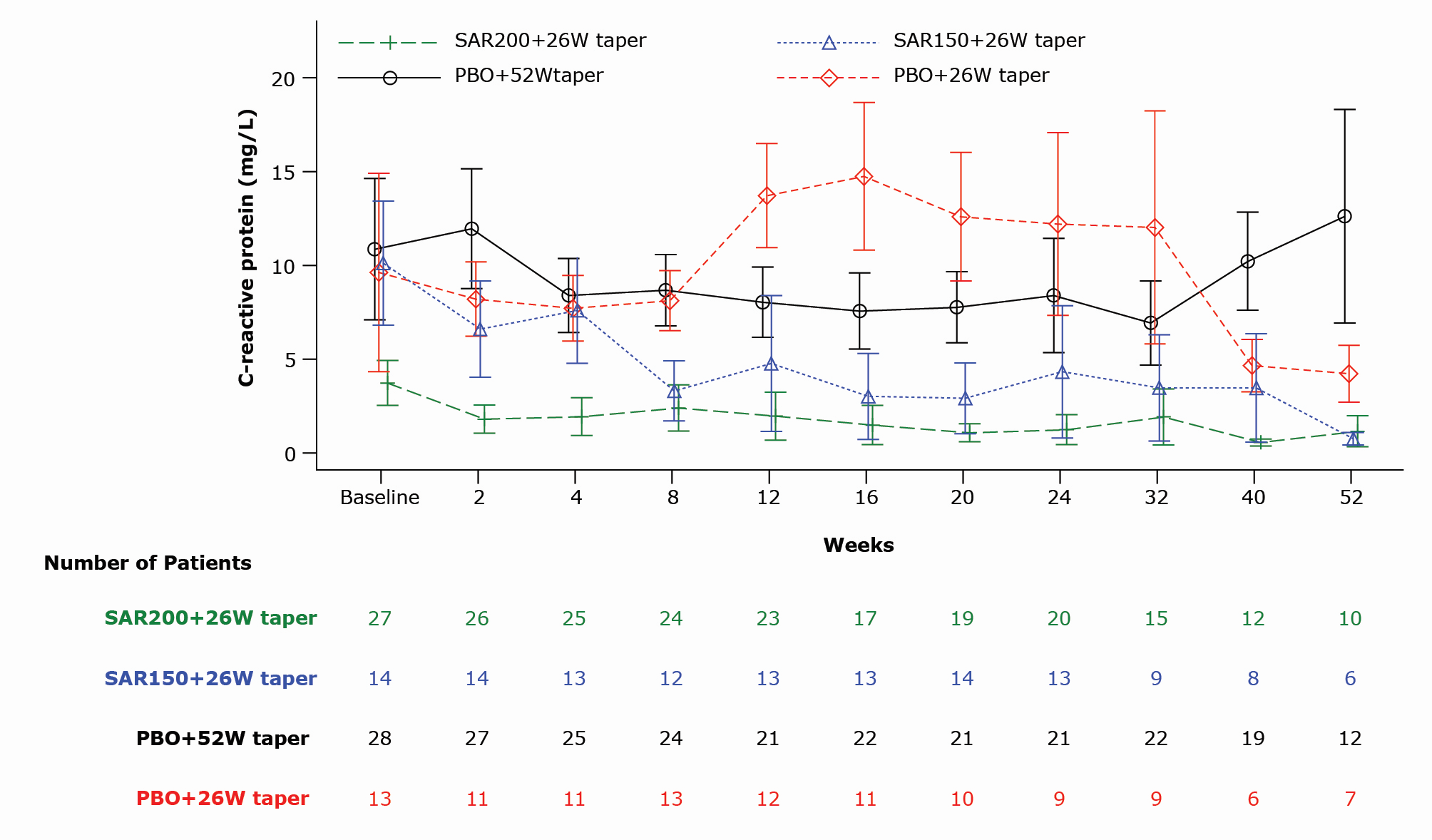


CRP, C-reactive protein; PBO, placebo; SAR150/200, sarilumab 150/200 mg; TEAE, treatment-emergent adverse event; W, week
