# Supplementary material for: A phase 3 randomized, double-blind, placebo-controlled study to evaluate the efficacy and safety of sarilumab in patients with giant cell arteritis
Source: Arthritis Res Ther. 2023 Oct 16;25:199. doi: 10.1186/s13075-023-03177-6 (PMC10577982; doi:10.1186/s13075-023-03177-6)
Supplement: Supplementary file 7 — Additional file 7: Fig. S3. Serum sarilumab trough concentrations during the TEAE period – PK population: a) Functional mean (SD) concentration over time; b) Functional geometric mean concentration over time. [file 13075_2023_3177_MOESM7_ESM.docx]

**Additional file 7**

**Fig. S3** Serum sarilumab trough concentrations during the TEAE period – PK population: a) Functional mean (SD) concentration over time; b) Functional geometric mean concentration over time


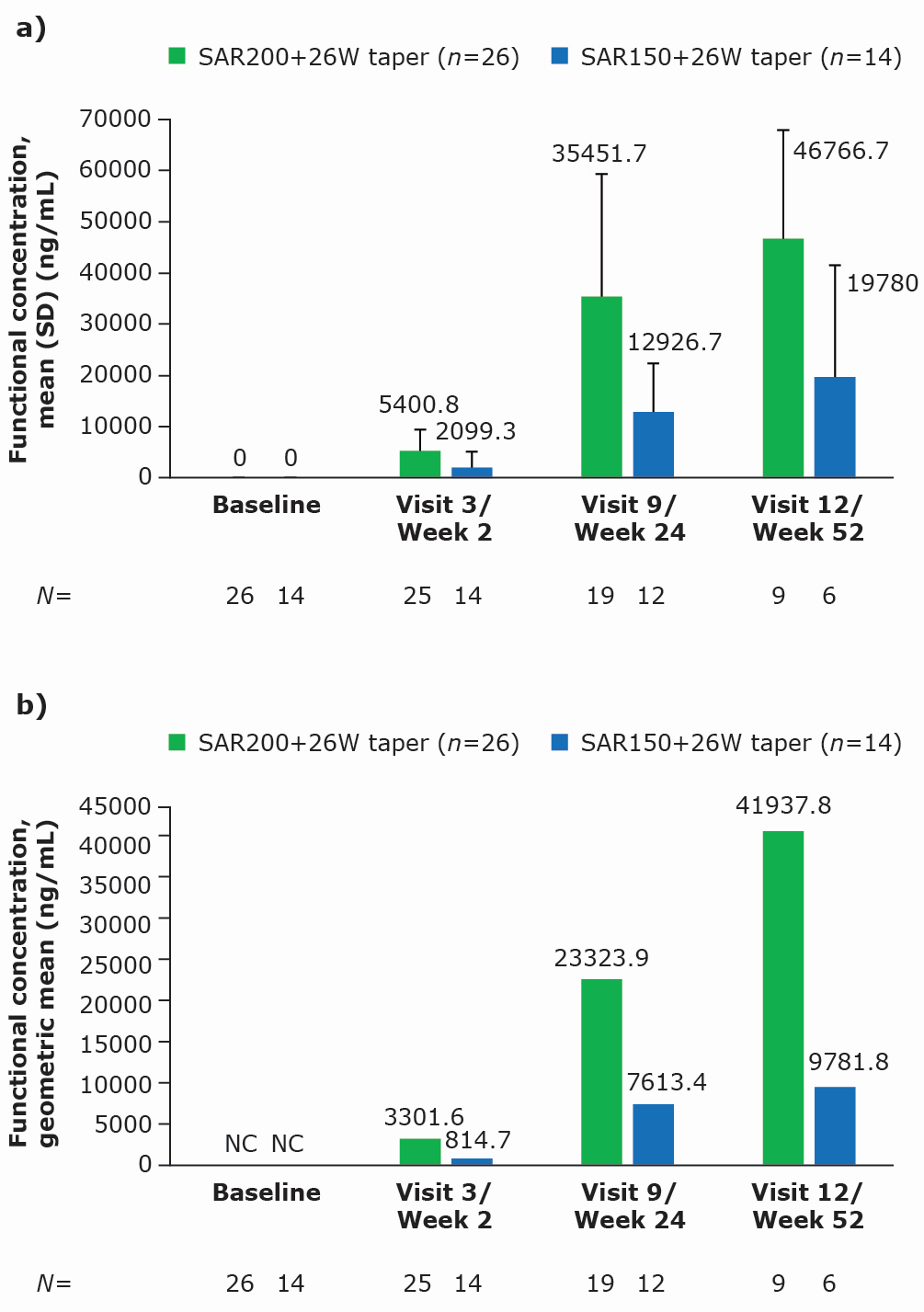


*N*, number of patients; NC, not calculated; PK, pharmacokinetics; SAR150/200, sarilumab 150/200 mg; SD, standard deviation; TEAE: treatment-emergent adverse event
